# Abundance of Colistin-Resistant, OXA-23- and ArmA-Producing *Acinetobacter baumannii* Belonging to International Clone 2 in Greece

**DOI:** 10.3389/fmicb.2020.00668

**Published:** 2020-04-15

**Authors:** Mattia Palmieri, Marco Maria D’Andrea, Andreu Coello Pelegrin, Nadine Perrot, Caroline Mirande, Bernadette Blanc, Nicholas Legakis, Herman Goossens, Gian Maria Rossolini, Alex van Belkum

**Affiliations:** ^1^bioMérieux, Data Analytics Unit, La Balme-les-Grottes, France; ^2^Department of Medical Biotechnologies, University of Siena, Siena, Italy; ^3^Department of Biology, University of Rome Tor Vergata, Rome, Italy; ^4^bioMérieux, R&D Microbiology, La Balme-les-Grottes, France; ^5^Central Laboratories, IASO Group Hospitals, Athens, Greece; ^6^Laboratory of Medical Microbiology, Vaccine and Infectious Disease Institute, University of Antwerp, Antwerp, Belgium; ^7^Department of Experimental and Clinical Medicine, University of Florence, Florence, Italy; ^8^Clinical Microbiology and Virology Unit, Florence Careggi University Hospital, Florence, Italy

**Keywords:** colistin resistance, Greece, *A. baumannii*, genomics, *pmrCAB*, lipid A

## Abstract

Carbapenem resistant *Acinetobacter baumannii* (CRAB) represents one of the most challenging pathogens in clinical settings. Colistin is routinely used for treatment of infections by this pathogen, but increasing colistin resistance has been reported. We obtained 122 CRAB isolates from nine Greek hospitals between 2015 and 2017, and those colistin resistant (ColR; *N* = 40, 32.8%) were whole genome sequenced, also by including two colistin susceptible (ColS) isolates for comparison. All ColR isolates were characterized by a previously described mutation, PmrB^A226V^, which was associated with low-level colistin resistance. Some isolates were characterized by additional mutations in PmrB (E140V or L178F) or PmrA (K172I or D10N), first described here, and higher colistin minimum inhibitory concentrations (MICs), up to 64 mg/L. Mass spectrometry analysis of lipid A showed the presence of a phosphoethanolamine (pEtN) moiety on lipid A, likely resulting from the PmrA/B-induced *pmrC* overexpression. Interestingly, also the two ColS isolates had the same lipid A modification, suggesting that not all lipid A modifications lead to colistin resistance or that other factors could contribute to the resistance phenotype. Most of the isolates (*N* = 37, 92.5%) belonged to the globally distributed international clone (IC) 2 and comprised four different sequence types (STs) as defined by using the Oxford scheme (ST 425, 208, 451, and 436). Three isolates belonged to IC1 and ST1567. All the genomes harbored an intrinsic *bla*_OXA–51_ group carbapenemase gene, where *bla*_OXA–66_ and *bla*_OXA–69_ were associated with IC2 and IC1, respectively. Carbapenem resistance was due to the most commonly reported acquired carbapenemase gene *bla*_OXA–23_, with IS*Aba1* located upstream of the gene and likely increasing its expression. The *armA* gene, associated with high-level resistance to aminoglycosides, was detected in 87.5% of isolates. Collectively, these results revealed a convergent evolution of different clonal lineages toward the same colistin resistance mechanism, thus limiting the effective therapeutic options for the treatment of CRAB infections.

## Introduction

*Acinetobacter baumannii* is now recognized as a major hospital pathogen by its ability to resist major antimicrobials and to survive in the healthcare environment (Peleg et al., 2008). Currently, carbapenem resistant *A. baumannii* (CRAB) is widespread, with rates reaching or exceeding 90% in some clinical settings in Southern and Eastern European countries (ECDC, 2018) and elsewhere^1^, and mortality rates for the most common CRAB infections such as bloodstream infections and hospital acquired pneumoniae approaching 60% (Wong et al., 2017). OXA-type carbapenemases constitute the most prevalent mechanism of carbapenem resistance in this species, with OXA-23, OXA-24, and OXA-58 being the most prevalent enzymes (Poirel and Nordmann, 2006). Molecular epidemiological studies usually revealed an oligoclonal distribution of CRAB, with outbreak strains mostly belonging to international clones (IC) 1 and 2 (Zarrilli et al., 2013).

Since their first emergence in 2000, CRAB have become endemic, and the percentage of carbapenem resistance reached 94% in 2017 (Tsakris et al., 2003; ECDC, 2018). Regarding the CRAB clonal nature and carbapenemase gene content, a study conducted from 2000 to 2009 in Greece showed that CRAB were harboring only the OXA-58 carbapenemase gene; while IC1 was prevalent until 2004, IC2 became dominant during 2005–2009 (Gogou et al., 2011). Between 2009 and 2011, OXA-23 producers emerged and replaced the previously predominant OXA-58 producing *A. baumannii* strains (Liakopoulos et al., 2012). Recently, a molecular epidemiological study on contemporary CRAB clinical isolates derived from hospitals throughout Greece demonstrated the predominance of OXA-23 producers belonging to IC2 (Pournaras et al., 2017).

Colistin-based treatment often represents the only therapeutic option for CRAB infections (Viehman et al., 2014). However, CRAB isolates that are also colistin resistant (ColR) are being reported more frequently. Data from the EARS-Net study in 2016 collected from 30 European countries showed that 4.0% of the tested isolates were resistant to colistin, with the vast majority (70.7%) of the resistant isolates reported from Greece and Italy (ECDC, 2017). A study from Greece reported an increase in colistin resistance from 1% in 2012 to 21.1% in 2014 (Oikonomou et al., 2015), while Pournaras et al. (2017) reported a resistance rate of 27.3% in 2015. More alarmingly, the colistin resistance rate was 56.8% in isolates collected from patients with ventilator-associated pneumonia in Greece during 2015 (Nowak et al., 2017). Colistin resistance has been linked to mutations in the two-component transcriptional regulator genes *pmrA/B* and consequent *pmrC* overexpression in most instances. The phosphoethanolamine (pEtN) phosphotransferase PmrC adds a pEtN group to the lipid A of the lipopolysaccharide, lowering the net negative charge of the cell membrane, thus impacting the binding of colistin and preventing the cell membrane leakage (Poirel et al., 2017). Colistin resistance may also result from the overexpression of *etpA*, a *pmrC* homolog. This is mediated by insertional inactivation of a gene encoding an H-NS family transcriptional regulator (Lucas et al., 2018) or by integration of insertion sequence elements upstream of the *eptA* gene itself (Gerson et al., 2019; Potron et al., 2019; Trebosc et al., 2019).

In this study, 40 ColR and two colistin susceptible (ColS) CRAB isolates collected from nine Greek hospitals between 2015 and 2017 were studied. Whole genome sequencing was performed to investigate the mechanisms of antibiotic resistance as well as the genomic relatedness between the strains.

## Materials and Methods

### Bacterial Strains and Antimicrobial Susceptibility Testing

**FIGURE 1:**
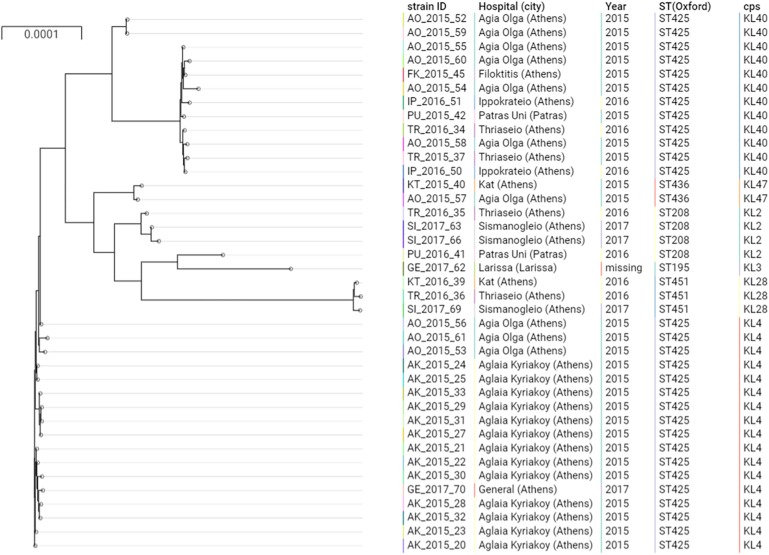
Phylogenetic tree of the *A. baumannii* clinical isolates belonging to IC2. Cps: capsular polysaccharides.

In the period 2015–2017, a total of 122 consecutive non-duplicate clinical CRAB isolates were obtained from routine microbiological cultures of clinical samples (e.g., urine, blood, skin, bronchial aspirate) from different patients admitted to nine Greek hospitals involved in this study (Figure 1). Bacteria were not isolated by the authors but provided by the respective medical centers. Therefore, an ethics approval was not required as per institutional and national guidelines and regulations. Antimicrobial susceptibility testing was performed using the Vitek2 instrument (bioMérieux, Marcy l’Étoile, France) and the results were interpreted following the EUCAST breakpoints (EUCAST, 2019). Since EUCAST doesn’t provide breakpoints for cephalosporins and *Acinetobacter* spp., CLSI breakpoints were used for those antibiotics (CLSI, 2019). Colistin minimum inhibitory concentrations (MICs) were obtained by broth microdilution following the CLSI guidelines (CLSI, 2019), and the results were interpreted following the EUCAST susceptibility breakpoint of 2 mg/L (EUCAST, 2019). Only the ColR CRAB isolates plus two randomly selected ColS CRAB isolates were retained for further experiments.

### Genome Sequencing and Assembly

Whole DNA of the selected CRAB isolates was extracted using the QIAGEN UltraClean Microbial kit and sequenced with a NovaSeq sequencer (Illumina, United States), generating paired end reads of 100 bp. Raw reads were assembled using SPAdes v.3.11.1 (Bankevich et al., 2012) and annotated with Prokka (Seemann, 2014). Whole genome sequencing data have been deposited under BioProject PRJNA578598.

### Bioinformatics Analysis

Sequence types (STs) were assigned by the mlst tool^2^ by using the Oxford (*gltA*, *gyrB*, *gdhB*, *recA*, *cpn60*, *gpi*, and *rpoD* genes) and the Pasteur (*cpn60*, *fusA*, *gltA*, *pyrG*, *recA*, *rplB*, and *rpoB* genes) schemes available on pubMLST.org. The ABRicate tool^3^ was used for the detection of antimicrobial resistance genes, by using the ResFinder (Zankari et al., 2012), CARD (Jia et al., 2017), BLDB (Naas et al., 2017), and ARG-ANNOT (Gupta et al., 2014) databases. The minimum percentage of coverage and identity used were 60 and 90%, respectively. The Kaptive tool was used to detect the KL and OC locus (Wyres et al., 2019). BLAST+ (2.7.1) was used to detect mutations in genes previously demonstrated to be potentially involved in colistin resistance (i.e., *pmrCAB*, *eptA*), and only those leading to amino acid variations were considered. The *pmrA/B/C* and *eptA* genes were compared to the reference genome ACICU (accession no. CP031380.1). The presence of insertion sequence elements in the 500 bp region upstream of the *bla*_ADC_, *bla*_OXA–23_, *armA*, *eptA*, and *pmrC* genes was determined using the ISfinder tool (Siguier et al., 2006). Core genes were defined by Roary (v3.12.0) (Page et al., 2015) by using the annotated genomes, and genomes belonging to different ICs were treated separately. The alignment of these genes was screened for further recombination using Gubbins (v2.3.4) (Croucher et al., 2015), while an ML phylogeny was obtained by using RAxML (v8.2.12) (Stamatakis, 2014) with the GTRGAMMA model and 100 bootstrap replicates. The phylogenetic tree was visualized together with associated metadata using Microreact (v7.0.0) (Argimón et al., 2016). Single nucleotide polymorphisms (SNPs) were obtained with the snp-dists tool^4^ by using the Roary core genes alignment as input.

### Analysis of Lipid A

Lipid A was extracted using an acetic acid-based procedure as previously described (Kocsis et al., 2017). Once extracted, 0.7 μL of the concentrate was spotted on a matrix-assisted laser desorption/ionization–time of flight mass spectrometry (MALDI-TOF MS) plate followed by 0.7 μL of norharmane matrix (Sigma-Aldrich, St Louis, MO, United States) and then air-dried. The samples were analyzed on a Vitek MS instrument (bioMérieux, Marcy l’Étoile, France) in the negative-ion mode. The resulting spectra were compared to that obtained for the ColS reference strain *A. baumannii* ATCC 19606.

## Results

### Bacterial Strains and Antimicrobial Susceptibilities

Of the 122 CRAB isolates, 40 (32.8%) were also ColR, with colistin MICs ranging from 4 to 64 mg/L. All following data concern only the ColR isolates. Antimicrobial susceptibility testing revealed that all isolates were resistant to cephalosporins (ceftazidime and cefepime), carbapenems (imipenem and meropenem) fluoroquinolones (ciprofloxacin and levofloxacin) and tobramycin. Resistance rates for gentamicin and trimethoprim/sulfamethoxazole were 87.5% (*N* = 35) and 92.5% (*N* = 37), respectively. The two ColS CRAB isolates, included in this study for comparative purposes, had a colistin MIC of 0.5 mg/L (Supplementary Table S1).

### Genomic Epidemiology

The majority of the ColR CRAB isolates (*N* = 37, 92.5%) were ST2, belonging to the previously described IC2 as defined by the Pasteur MLST scheme (Diancourt et al., 2010; Figure 1). The Oxford MLST scheme allowed to further differentiate the IC2 isolates in 4 different STs, all belonging to the clonal complex (CC) 208: the majority of isolates (*N* = 29, 78.4%) belonged to ST425, while ST208, ST451 and ST436 represented the 8.1% (*N* = 3), 8.1% (*N* = 3) and 5.4% (*N* = 2), respectively. These four STs shared six out of seven alleles and differed only by the *gpi* gene. The *gpi* gene is one of the capsular polysaccharide synthesis genes; therefore, the Oxford MLST scheme suffers from limitations, as the *gpi* gene is prone to homologous recombination (Gaiarsa et al., 2019). From a total of 4,612 different genes detected in all the isolates, 3,031 (65.7%) were core genes. Core gene SNPs among IC2 genomes varied between 2 and 1,652 (mean: 569, median: 531).

The phylogenetic analysis of IC2 isolates shows two major clusters of ST425, well differentiated in the tree. These two clusters were characterized by two different capsular polysaccharides, KL4 and KL40.

Different capsular polysaccharides were observed in the other IC2 isolates (Figure 1), while all the IC2 isolates were characterized by the lipooligosaccharide outer core (OC) locus 1 (OCL1).

Isolates of ST425:KL4 were only observed in Athens, within two hospitals in 2015 (Aglaia Kyriakoy and Agia Olga) and one isolate in the Thriassio General hospital in 2017. The 13 isolates obtained from the Aglaia Kyriakoy hospital had an average of 12 core SNPs, suggesting cross-transmission of isolates between different patients. Isolates of ST425:KL40 were retrieved between 2015 and 2016 from four hospitals in Athens and one isolate from the University Hospital in Patras (200 km west of Athens). This underscores the endemicity at the local level of this clone, moreover, suggesting inter-hospital cross infections, given the absence of a clear clustering in the tree of isolates obtained from different hospitals.

The remaining three isolates belonged to ST1 (IC1) and ST1567 according to the Pasteur and Oxford MLST schemes, respectively, and harbored a capsule and lipooligosaccharide of type KL40 and OCL2. Core gene SNPs varied between 29 and 305.

The two ColS CRAB isolates belonged to IC2, or ST208 (isolate PU_2016_41) and ST195 (GE_2017_62) by using the Oxford MLST scheme, and had a median of 706 (min: 8, max: 1,568) and 1175 SNPs (min: 725, max: 1,652) compared to the ColR isolates, respectively.

### Colistin Resistance Mechanisms

Several chromosomal mutations in genes potentially involved in colistin resistance were detected, in comparison with the ACICU ColS reference genome. The mutation PmrB^A138T^ was detected in all ColR and ColS isolates, indicating that it may not contribute significantly to the resistance phenotype, as previously reported (Oikonomou et al., 2015). Conversely, the mutation A226V in the histidine kinase A (phosphoacceptor) domain of PmrB was observed in all ColR isolates, and not in the ColS ones (Figure 1). This mutation has been described in several prior studies, associated with ColR strains (Arroyo et al., 2011; Mavroidi et al., 2015, 2017; Dortet et al., 2018; Trebosc et al., 2019).

Isolates with PmrB^A226V^ without other alterations had colistin MICs ranging from 4 to 8 mg/L. When an additional mutation in PmrB occurred (PmrB^E140F^ in AK_2015_33 and PmrB^L178F^ in SI_2017_69), strains showed a colistin MIC of 16 mg/L. Two strains belonging to IC1 (FK_2016_46 and FK_2016_47) had an additional K172I mutation in the transcriptional regulatory protein C-terminal domain of PmrA, and showed colistin MICs of 32 mg/L. Finally, the strain AO_2015_54 had an additional D10N mutation in the CheY-homologous receiver PmrA domain and was associated with colistin MIC of 64 mg/L. All these additional mutations are, to the best of our knowledge, first described here.

The susceptible strain GE_2017_62 had no additional mutations in *pmrA/B* genes. However, it had an IS*Aba1* positioned 110 bp upstream of the *pmrC* gene, in reverse orientation. This is, to the best of our knowledge, the first report of an insertion sequence transposition upstream of the *pmrC* gene. However, this transposition event doesn’t seem to alter the colistin susceptibility in this isolate. The second susceptible strain PU_2016_41 had PmrA^M12V^ and PmrB^R181H+Y388N^. These mutations are firstly described here, and in this strain they don’t seem to impact the colistin susceptibility.

The *pmrC* homolog *eptA* was detected in all the isolates of the IC2 except the susceptible isolate GE_2017_62, while it was absent in the IC1 isolates. The obtained *eptA* gene sequences were identical to that of the susceptible reference ACICU, and did not present insertion elements in the upstream region.

The *mcr* genes, encoding for acquired colistin resistance, have not been described in *A. baumannii* yet, and were not detected in our strain collection.

### Lipid A Modifications

An increased expression of *pmrC* or *eptA* results to the addition of pEtN to lipid A. The lipid A of the ColR and ColS CRAB isolates was extracted and analyzed by MALDI-TOF MS, and the resulting spectra were compared to that of the ColS reference strain *A. baumannii* ATCC 19606.

**FIGURE 2:**
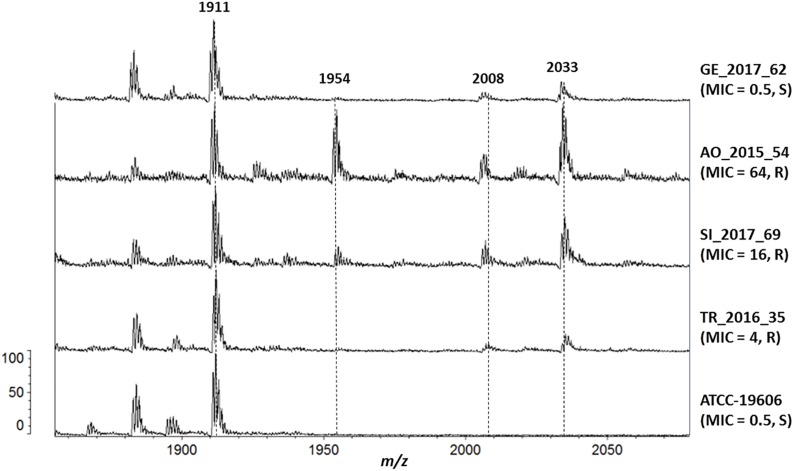
Mass spectrometry analysis of Lipid A. From the bottom, isolate (colistin MIC, resistant/susceptible): ATCC-19606 (0.5, S), TR_2016_35 (4, R), SI_2017_69 (16, R), AO_2015_54 (64, R) and GE_2017_62 (0.5, S).

Several lipid A species were detected in the reference strain ATCC 19606 and in all clinical isolates: hepta-acylated lipid A (*m*/*z* 1,910), hexa-acylated lipid A (*m*/*z* 1,728) and tetra-acylated lipid A (*m*/*z* 1,404). The addition of pEtN (*m*/*z* 124) to lipid A was shown by the mass at *m*/*z* 2,034, and unexpectedly it was observed in all the clinical strains, including the ColS ones (Figure 2).

Isolates with colistin MICs higher than 8 mg/L also showed the peak at *m*/*z* 1,954, representing the pEtN-modified hepta-acylated lipid A (*m*/*z* 2,034) minus one phosphate group (*m*/*z* 80), as previously reported (Kim et al., 2014).

The addition of galactosamine to lipid A, which is indicated by a mass at *m*/*z* 2,071 (Pelletier et al., 2013), was not observed in any isolate.

### Antimicrobial Resistance Mechanisms and Phenotype Correlation

All CRAB genomes harbored a chromosomal *bla*_ADC_ cephalosporinase, an intrinsic *bla*_OXA–51_ group carbapenemase and an acquired *bla*_OXA–23_. IC2 genomes contained the *bla*_ADC–73_ (accession no. KP881233), a variant of *bla*_ADC_ with a sequence identity of 1,151/1,152 nucleotides compared to that of *bla*_ADC–30_, and previously observed in IC2 isolates (Karah et al., 2016). An IS*Aba1* element was present 9 bp upstream of the *bla*_ADC–73_ gene in reverse orientation in all IC2 genomes, and it is responsible to increase the cephalosporinase gene expression (Héritier et al., 2006). Conversely, IC1 genomes contained *bla*_ADC–175_ (MH594297) with an IS*Aba125* element positioned 66 bp upstream the gene in reverse orientation, as also previously reported (Lopes and Amyes, 2012). IS*Aba125* was shown to increase the cephalosporinase expression 6 times more than IS*Aba1* (Lopes and Amyes, 2012). The allelic variants of the intrinsic *bla*_OXA–51_-like carbapenemase genes were *bla*_OXA–66_ and *bla*_OXA–69_, associated with IC2 and IC1, respectively, as previously observed (Zander et al., 2012). All the *bla*_OXA–23_ genes were characterized by the presence of an IS*Aba1* located upstream of the gene, which has been previously demonstrated to increase its expression (Turton et al., 2006). In particular, the *bla*_OXA–23_ gene was part of a Tn*2006* transposon in the IC2 genomes. Conversely, a Tn*2008* embedded within a Tn*aphA6* was found in the three IC1 genomes, matching 100% with the sequence of plasmid pABKp1 (KP074966.1) obtained from *A. baumannii* isolates from Romania (Gheorghe et al., 2014). Consistently with the mentioned genes and their genetic environments, all isolates were resistant to cephalosporins, including ceftazidime (3rd generation) and cefepime (4th generation), and carbapenems (imipenem and meropenem).

Aminoglycoside resistance genes were observed among the isolates, namely *aac(3)-I*, *aac(3)-Ia*, *ant(3″)-1a*, *aph(3′)-Ia*, *aph(3′)-Via*, *aph(6)-Id*, *armA*, and *strA* (Supplementary Table S1). ArmA is a 16S ribosomal RNA methyltransferase, which protects the 30S ribosomal subunit from aminoglycoside binding and conferring high aminoglycosides MICs. Consistently, all the strains carrying *armA* (35/40, 87.5%) were resistant to gentamicin and tobramycin. The *armA* gene was located in the chromosome aboard on the widely disseminated Tn*1548*, and it was found downstream of a cluster of genes encoding proteins annotated as paraquat-inducible protein A and protein B, as previously described for ST195 strain AC29 (Lean et al., 2016).

All strains contained substitutions within the QRDR, namely GyrA^S83L^ and ParC^S80L^, previously associated to quinolone resistance (Vila et al., 1995, 1997). As expected, all strains were non-susceptible to ciprofloxacin and levofloxacin.

## Discussion

Carbapenems represent first-line agents for the treatment of *A. baumannii* infections, consequently the rise of infections due to carbapenem-resistant strains is of major concern. The carbapenem resistance in the isolates described here was associated to the IS*Aba1*-mediated overexpression of *bla*_OXA–23_ located either in Tn*2006* (IC2 isolates) or Tn*2008* (IC1) transposons. Previous studies reported that OXA-23 producers emerged and replaced the previously predominant OXA-58 *A. baumannii* isolates (Liakopoulos et al., 2012), and this phenomenon could be linked to the stronger hydrolytic activity of OXA-23 compared to OXA-58 (Peleg et al., 2008).

Additionally, most CRAB isolates are susceptible to only 1 or 2 agents, making them extensively drug-resistant (XDR) pathogens (Viehman et al., 2014).

Because of the increasing use of colistin, resistance to this antibiotic has rapidly increased, especially in CRAB isolates (Giamarellou, 2016; Jeannot et al., 2017), and now reached critical levels in some countries (Nowak et al., 2017). From the nine hospitals involved in this study, the 32.8% of the CRAB isolates were also ColR. These results indicate that colistin resistance rates among CRAB isolates from Greece is on the rise, as a previous study reported a resistance rate of 27.3% in 2015 (Pournaras et al., 2017). While the *mcr* genes, encoding for acquired colistin resistance, were absent among our isolates, we found several mutations in the *pmrCAB* operon associated with the colistin resistance phenotype. Interestingly, the previously described PmrB^A226V^ mutation, previously associated to low-level colistin resistance, was detected in all the ColR isolates but no in the ColS ones. In a recent study, Trebosc et al. (2019) investigated the colistin resistance mechanisms of 12 clinical *A. baumannii* strains. The authors concluded that colistin resistance was conferred, in most cases, by mutations in the PmrB sensor kinase that led to PmrC overexpression. Two of those strains were isolated in Greece in 2012, belonged to either IC1 or IC2 and had the mutation PmrB^A226V^. Such findings support the important role of the mentioned PmrB mutation in the colistin resistance phenotype. Moreover, a similar substitution of the alanine in position 226 of PmrB was reported to confer stable colistin resistance in clinical *A. baumannii* isolates (Charretier et al., 2018). Some of our isolates had additional mutations in either PmrB or PmrA, and were associated with higher colistin MICs, up to 64 mg/L. Multiple mutations may result in an increased expression of *pmrC*, as recently shown by RNA-Seq experiments (Wright et al., 2017) and by qRT-PCR (Gerson et al., 2020). However, the same studies reported clinical isolates characterized by *pmrC* overexpression due to PmrA/B mutations, but with an unexpected ColS phenotype. Similarly, the two ColS isolates from our study had *pmrCAB* alterations and a modified lipid A, as observed with mass spectrometry. All these observations support the hypothesis that additional and still unknown factors are involved in colistin resistance of clinical *A. baumannii* isolates (Jeannot et al., 2017; Gerson et al., 2019, 2020). Determination of the cell-envelope charge could be useful in the elucidation process of the complex mechanism of colistin resistance in *A. baumannii* (Cafiso et al., 2019).

In this study, we observed a clear predominance of IC2, which is globally distributed (Higgins et al., 2010) and which is gradually replacing IC1 (Gogou et al., 2011; Villalon et al., 2011). The major sequence type within IC2 was ST425, as defined by the Oxford MLST scheme. To the best of our knowledge, only one study reported such ST, with one clinical isolate collected in 2002 in Sydney, Australia (Nigro and Hall, 2016). However, WGS data were not provided. Both capsular polysaccharides reported within our ST425 isolates, KL4 and KL40, were rarely observed (0.2%) or completely absent, respectively, within IC2 genomes in a recent study where 3,416 publicly available *A. baumannii* genomes were analyzed (Wyres et al., 2019). Conversely, KL4 and KL40 represented the second (20.1%) and third (11.9%) most common capsular types observed within IC1 genomes. It is conceivable that ST425 resulted from homologous recombination between a CC208 and an IC1 genomes, where the IC1 capsular polysaccharides genes were acquired by the CC208 strain, as this region was previously shown to be a frequent subject of homologous recombination (Adams et al., 2008; Snitkin et al., 2011; Kenyon and Hall, 2013).

## Conclusion

Genomic analysis of ColR CRAB isolates from different Greek hospitals revealed a convergent evolution of different clonal lineages toward the same colistin resistance mechanism, characterized by the mutation PmrB^A226V^. The prevalence of ColR CRAB isolates belonging to IC2 and expressing OXA-23 and ArmA is increasing, and it represents a huge threat within clinical settings, given the very limited effective agents for the treatment of infections caused by such strains.

## Data Availability Statement

The datasets generated for this study can be found in the PRJNA578598.

## Ethics Statement

Ethical review and approval was not required for the study on human participants in accordance with the local legislation and institutional requirements. Written informed consent for participation was not required for this study in accordance with the national legislation and the institutional requirements.

## Author Contributions

AB, GR, and HG conceived and designed the study. MP and NP performed the phenotypic and genomics experiments. MP and MD’A performed the bioinformatics analysis. All authors analyzed the data and contributed to the manuscript.

## Conflict of Interest

NL was employed by IASO Group Hospitals. The remaining authors declare that the research was conducted in the absence of any commercial or financial relationships that could be construed as a potential conflict of interest.

## Footnotes

1 https://resistancemap.cddep.org

2 https://github.com/tseemann/mlst

3 https://github.com/tseemann/abricate

4 https://github.com/tseemann/snp-dists

## Supplementary Material

TABLE S1 Metadata, genotypic and phenotypic results of the 42 CRAB from Greece.
